# An explainable machine learning analysis of technical and tactical indicators associated with CSL match outcomes

**DOI:** 10.3389/fpsyg.2026.1854812

**Published:** 2026-06-24

**Authors:** Shihuan Chen, Xuewei Li, Yan Ouyang, Wei Hong, Weilang Jiang, Fangqing Li, Ying Zhao, Ying Liu, Yanyi Zhao, Tong Zhou

**Affiliations:** 1 School of Intelligent Sports Engineering, Wuhan Sports University, Wuhan, Hubei, China; 2 Football Performance Insight Laboratory, Wuhan Sports University, Wuhan, Hubei, China; 3 School of Physical Education, South China Normal University, Guangzhou, Guangdong, China; 4 Wuhan Three Towns Football Club, Wuhan, Hubei, China

**Keywords:** applied sports science, CSL, explainable machine learning, football performance analysis, SHAP

## Abstract

To examine the analytical value of football technical and tactical indicators in post-match outcome analysis, this study used post-match data from 240 matches in the 2024 Chinese Super League (CSL) season and constructed an explainable analytical framework integrating machine learning and SHAP (Shapley Additive Explanations). From the home-team perspective, this study aimed to use model comparison and SHAP-based interpretation to identify the relative contribution magnitude, class-specific contribution direction, and potential nonlinear contribution patterns of technical, tactical, contextual, and player-attribute indicators in the classification of home win, draw, and home loss. Based on indicator attributes and the logic of football match performance, the variables were classified into seven dimensions: player attributes, match context, attacking performance, possession and passing performance, duels and contests, defensive performance, and disciplinary performance. Multiple machine learning models were compared in the three-class match outcome classification task, and XGBoost was used as the base model for subsequent SHAP analysis because of its comparatively balanced validation performance and tree-based interpretability. The global SHAP results showed that xG and Value had the highest model contributions, indicating that chance quality and squad-resource-related information were the primary signals used by the model to distinguish match outcome classes. Yellow_C, Round, Long_B, Throw_ins, Blocked_S, GK_Keep_Save, and Recoveries also ranked highly, suggesting that match outcome classification was informed by multidimensional information related to match context, possession progression, defensive events, goalkeeper involvement, and disciplinary performance. The class-specific SHAP results showed that xG and Value exhibited opposite contribution directions between the home win and home loss classes, whereas SHAP values for the draw class were more concentrated around zero, indicating weaker dominance of individual variables in identifying draw outcomes. The SHAP dependence plots further showed that several variables exhibited potential nonlinear contribution patterns and model-derived transition points in the home win class. Overall, this study provides an explainable post-match analytical framework for identifying how technical, tactical, contextual, and player-attribute indicators contribute to CSL match outcome classification, offering data-driven reference for contextual video review, performance diagnosis, and training-priority development.

## 1 Introduction

In recent years, with the development of big data and artificial intelligence technologies, data analytical methods have been increasingly applied in the field of sports (Cao, 2023). Football match analysis has also gradually shifted from traditional descriptive statistics and difference testing toward machine learning-based complex data modeling. Algorithms such as random forests, support vector machines, and logistic regression have been applied to football match outcome analysis and have shown certain advantages in handling high-dimensional and nonlinear data structures (Bunker et al., 2024; Hassard and Kerr, 2024; Atta Mills et al., 2024). Among these methods, gradient boosting ensemble approaches have been widely used in studies of match outcome modeling, team performance evaluation, and the identification of key player performance indicators because of their strong feature representation capacity and classification performance (Moya et al., 2025; Almulla and Alam, 2020). Existing studies have shown that, compared with some traditional statistical models, ensemble learning models generally demonstrate better predictive performance and stability in football match outcome classification tasks (Hubáček et al., 2019; Berrar et al., 2019; Tax and Joustra, 2015).

However, solely pursuing model classification accuracy cannot fully meet the practical needs of post-match football analysis (Dixon and Coles, 1997; Hvattum and Arntzen, 2010). For coaches and match analysts, post-match review requires not only an understanding of the match outcome itself, but also a clear identification of which performance indicators the model mainly relies on when distinguishing different outcome classes, as well as the contribution direction of these indicators across different result categories. With the development of explainable artificial intelligence, the SHapley Additive exPlanations (SHAP) method can quantify the contribution of different variables to model output at both global and local levels, providing a relatively intuitive interpretive pathway for understanding complex models (Lundberg and Lee, 2017; Ribeiro et al., 2016; Bunker and Thabtah, 2019; Gong et al., 2022; McGarry et al., 2002; Hughes and Bartlett, 2002). Therefore, combining machine learning models with the SHAP method can help identify high-contribution performance indicators associated with match outcome classification from post-match data, and further explain the variable contribution structure underlying the model results.

In football matches, the final outcome is determined by the number of goals scored; however, match outcomes are not formed by a single technical or physical factor, but by the combined effects of multiple factors (Gong et al., 2022). From the perspectives of complex systems and match performance analysis, factors such as players’ technical performance, tactical execution, opposition quality, match status, and home–away context are interrelated, making match outcome analysis clearly context-dependent and nonlinear in nature (McGarry et al., 2002; Hughes and Bartlett, 2002; Carling et al., 2007; Rein and Memmert, 2016). With the development of match data availability and modeling approaches, an increasing number of studies have used statistical models and machine learning methods to analyze football match outcomes. However, many existing studies still primarily aim to improve the predictive accuracy of win–draw–loss outcomes or goal counts, with insufficient attention paid to the feature contributions, contribution directions, and value-range-specific changes that models rely on when distinguishing different match outcomes (Gudmundsson and Horton, 2017; Du et al., 2025; Myers, 2012). This prediction-performance-oriented research tendency may, to some extent, limit the translation of model results into post-match review and training practice.

Based on post-match data from the 2024 Chinese Super League, this study constructed an interpretable analytical framework integrating machine learning and the SHAP method. Specifically, this study aimed to use model comparison and SHAP-based interpretation to identify the relative contribution magnitude, class-specific contribution direction, and potential nonlinear contribution patterns of different indicators in the classification of home win, draw, and home loss. Furthermore, this study developed an indicator framework based on dimensions including player attributes, match context, attacking performance, possession and passing performance, duels and contests, defensive performance, and disciplinary performance. The findings aim to provide data-driven reference for post-match review, performance diagnosis, and training-priority development in the CSL.

## 2 Related research

Artificial intelligence technologies have been increasingly applied in sports event analysis, and machine learning methods have been used for tasks such as match outcome modeling, team performance evaluation, and the identification of key performance indicators (Xiang et al., 2024). Related studies have not been limited to football, but have also extended to sports such as basketball, baseball, and American football. Support vector machines, random forests, decision trees, and ensemble learning models have been applied to outcome classification and performance analysis in different competitive contexts (Rodrigues and Pinto, 2022; Ötting, 2021; Yaseen et al., 2022; Çene, 2022). Cai et al. (2019) constructed a hybrid ensemble learning framework based on CBA team match statistics, Jain and Kaur (2017) compared different machine learning models using NBA regular-season data, and Pai et al. (2017) further combined support vector machines with decision trees for basketball match outcome analysis.

In the field of football, a substantial body of research has been developed on match outcome modeling. These foundational studies have mainly demonstrated the applicability of machine learning to football match outcome analysis from the perspective of model performance. Rodrigues and Pinto (2022) used data from multiple seasons of the English Premier League to compare the performance of several models, including Naive Bayes, k-nearest neighbors, random forests, support vector machines, and artificial neural networks, in predicting match outcomes, and further discussed the practical value of these models by incorporating betting data.

Pugsee and Pattawong used a random forest model to predict football match outcomes, confirming the feasibility of traditional machine learning methods for this task (Pugsee and Pattawong, 2019). Stübinger and Mangold constructed models based on team- and player-level statistics from major European leagues, further indicating that structural information at both the team and player levels can provide complementary value for match outcome modeling (Stübinger et al., 2019).

In addition to model construction, match performance indicators themselves constitute an important component of football data analysis. Lago-Peñas et al. (2010) examined the relationships between technical statistics, such as ball possession and number of shots, and match outcomes from the perspective of contextual factors, including match location, opposition quality, and match status. They also identified key match statistics that could distinguish winning, drawing, and losing teams (Lago, 2009). Related studies have also indicated that distributional differences in indicators such as shooting, ball possession, passing, and defensive events across different match outcomes can provide a basis for post-match performance evaluation and technical–tactical analysis (Lago-Peñas et al., 2010). With the accumulation of event data and high-dimensional match data, the research perspective has gradually expanded from single technical statistics to cooperative structures within the match process. Lee constructed a graph-based model using team passing networks to examine the association between player cooperation patterns and match outcomes (Lee et al., 2025); Clemente et al. (2015) used complex network metrics to characterize the overall cooperative features of teams. Pena and Touchette (2012) analyzed passing structures and tactical organization in football from the perspective of network theory. Football match outcomes are not only related to individual technical statistics, but are also closely associated with match context, team cooperation, and tactical structure.

Existing studies have provided an important foundation in terms of model construction, algorithm comparison, and match performance indicator analysis. However, one limitation remains: most studies have placed greater emphasis on model predictive accuracy or differences in individual indicators, while paying insufficient attention to the variable contributions, contribution directions, and nonlinear changes that models rely on when distinguishing different match outcomes. For coaches and match analysts, the value of post-match review lies not only in judging the match result, but also in further understanding which performance indicators make relatively high contributions in the model and how these indicators are associated with the classification of win, draw, and loss outcomes. Based on this, using post-match data from the 2024 Chinese Super League, this study introduced the SHAP explainability method after comparing multiple machine learning models. It aimed to identify high-contribution performance indicators associated with the classification of home win, draw, and home loss, and to describe their class-specific contribution directions and potential nonlinear contribution patterns, thereby providing data-driven reference for post-match review and training-priority development.

## 3 Materials and methods

### 3.1 Dataset

We conducted a retrospective observational analysis of the Chinese Super League (CSL), which consists of 16 teams competing in a double round-robin format, with each team playing one home and one away match against every other team, resulting in 30 rounds and 240 matches in total. After comparing several open-access football data platforms, we selected publicly available match data from SofaScore as the data source for this study. SofaScore uses data provided by Opta Stats Perform, London, United Kingdom. The study by Liu et al. (2013) evaluated the inter-operator reliability of the company’s tracking system, and the results indicated acceptable reliability. The study showed excellent agreement between independent operators when coding team match events, with weighted kappa values of 0.92 and 0.94, respectively, and a mean event-timing difference of only 0.06 ± 0.04 s. The data collected in the present study covered matches from the 2024 season. The original dataset contained 188 feature variables, including multidimensional information such as basic match information, starting formations, post-match team ratings, and technical and tactical statistics.

To verify the reliability and validity of the website-derived dataset, a subsample of 16 matches was randomly selected across different rounds. Five professional football players with national first-class athlete qualifications reviewed the match videos and compared the observed match information with the data collected from SofaScore. The high intraclass correlation coefficient (ICC = 0.98) indicated a high level of agreement between the video-based verification and the website-derived data, supporting the reliability of the dataset.

This study used publicly available CSL match-level technical statistics from SofaScore. No human participants were recruited as research subjects, no human or animal experiments were conducted, and no intervention was performed. The expert video-based verification was conducted solely to assess the reliability of the website-derived match statistics, and no personally identifiable or sensitive information was collected. Therefore, informed consent and ethical approval were not required. The study was conducted in accordance with the relevant ethical principles of the Declaration of Helsinki.

### 3.2 Research design

By comparing multiple machine learning classification models, this study identified a suitable base model for SHAP-based post-match interpretation. The analytical focus was placed on how technical, tactical, contextual, and player-attribute indicators contributed to the classification of CSL match outcomes, including their relative contribution magnitude, class-specific contribution direction, and value-range-specific nonlinear patterns. Match outcome was modeled as a three-class classification task from the home-team perspective, with home win, draw, and home loss coded as 2, 1, and 0, respectively.

### 3.3 Experimental process

#### 3.3.1 Data processing

During data preprocessing, the original variables were first checked for missing values. Based on the missing-data pattern, 15 features with high missing rates were removed, mainly involving certain goalkeeper-save and key-error-related indicators. To reduce redundancy between corresponding home- and away-team technical statistics and to better capture the relative differences between the two competing teams on the same indicators, this study calculated difference variables between the home and away teams for corresponding features. Existing football performance analysis studies have also commonly compared match statistics between winning and losing teams, or between home and away teams, to identify key performance indicators associated with match outcomes (Lago-Peñas et al., 2010; Liu et al., 2015; Castellano et al., 2012).

Based on the attributes of the indicators and the logic of football match performance, the variables were classified into several dimensions, including player attributes, match context, attacking performance, possession and passing performance, duels and contests, defensive performance, and disciplinary performance (Hassard and Kerr, 2024; Hughes and Bartlett, 2002; Carling et al., 2007; Dormann et al., 2013). Considering that some technical and tactical indicators may be strongly correlated, VIF-based collinearity diagnostics were further conducted during the subsequent model training stage. Features with VIF values greater than 10 were removed, and the final retained variables were determined by combining collinearity diagnostics with domain knowledge and the interpretability of the variables (Dormann et al., 2013; Kutner et al., 2004). The specific variable names and definitions are presented in Table 1.

**Table 1:** Feature names.

Feature	Meaning	Category
Passes	Total passes	Possession/Passing
Long_B	Total long passes	Possession/Passing
Shots_off_T	Shots off target	Attacking Performance
Shots_O_Box	Shots on target	Attacking Performance
Clearances	Total clearances	Defensive Performance
A_Passes	Accurate passes	Possession/Passing
Long_B_S	Successful long passes	Possession/Passing
Final_T_E	Entries into final third	Possession/Passing
Tackles	Tackles	Duels/Contests
Corner_K	Corners	Match Context
Blocked_S	Blocked shots	Defensive Performance
Goal_K	Goal kicks	Match Context
GK_Keep_Save	Goalkeeper saves	Defensive Performance
Crosses_S	Successful crosses	Possession/Passing
Recoveries	Ball recoveries	Duels/Contests
Dispossessed	Times dispossessed	Duels/Contests
xG	Expected goals	Attacking Performance
Big_C_M	Big chances missed	Attacking Performance
Throw_ins	Throw-ins	Match Context
Fouled_in_final_third	Fouled in final third	Match Context
Fouls	Fouls committed	Discipline
Value	Player market value	Player Attributes
Interceptions	Interceptions	Defensive Performance
Dribbles_S	Successful dribbles	Attacking Performance
Offsides	Offsides	Discipline
Height	Player height	Player Attributes
Aerial_D	Successful aerial duels	Duels/Contests
Yellow_C	Yellow cards	Discipline
Red_C	Red cards	Discipline
Hit_Ww	Shots hitting woodwork	Attacking Performance
Through_B	Through balls	Possession/Passing
Tackles_W	Successful tackle rate	Duels/Contests
Year	Age	Player Attributes
Round	Match round	Match Context
Foreign	Number of starting foreign players	Player Attributes

Except for Round, all features were calculated as home-team values minus away-team values for the corresponding indicators.

#### 3.3.2 Model training and evaluation

During data processing, the full sample was stratified according to match outcome categories and divided into a training set and a held-out validation set at an 80:20 ratio. The training set contained 192 matches and was used for feature selection, model training, hyperparameter search, cross-validation, and model selection, whereas the held-out validation set contained 48 matches and was used only for final model performance evaluation. It did not participate in any feature selection, parameter optimization, or model training process.

**Figure 1:**
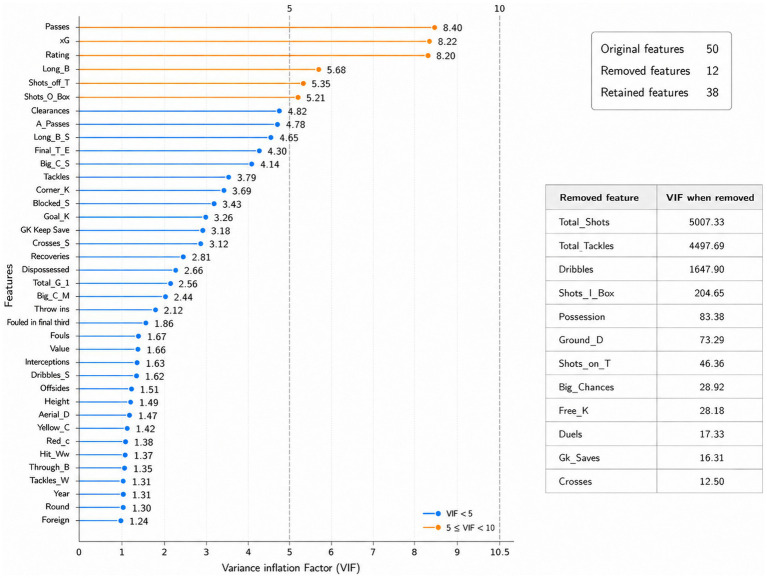
VIF-based collinearity diagnostics and feature screening results. Bars show the variance inflation factor values of retained features, and the dashed vertical lines indicate VIF thresholds of 5 and 10. The inset summarizes the number of original, removed, and retained features and lists the removed features with their corresponding VIF values.

Within the training set, stratified 10-fold cross-validation was used to evaluate the classification performance of each model, thereby reducing the influence of a single data split on model evaluation (Kohavi, 1995). For models involving missing-value imputation and feature standardization, SimpleImputer and StandardScaler were incorporated into a Pipeline, so that their parameters were fitted only within each training fold and then applied to the corresponding validation fold, thereby preventing information from the validation fold from entering the model training process (Pedregosa et al., 2011). VIF-based collinearity diagnostics were conducted within the training set, with features showing VIF values greater than 10 removed. The final retained variables were determined by combining the interpretability of the variables with domain knowledge (Dormann et al., 2013; Kutner et al., 2004). Model tuning was performed using RandomizedSearchCV and Optuna, both of which were conducted entirely within the training set (Bergstra and Bengio, 2012; Akiba et al., 2019). After the final model was refitted on the training set, its performance was evaluated using the held-out validation set (Figure 1).

To compare the classification performance of different models on the present dataset and to select an appropriate base model for subsequent interpretability analysis, seven machine learning models were compared: XGBoost, LightGBM, random forest, logistic regression, support vector machine, k-nearest neighbors, and decision tree (Breiman, 2001; Chen and Guestrin, 2016; Ke et al., 2017). Model performance was evaluated using Accuracy, Precision_macro, Recall_macro, F1_macro, AUC-OVR, and LogLoss. Considering performance on the held-out validation set, cross-validation stability, and suitability for subsequent interpretation, the model with relatively stable performance was selected for SHAP-based feature interpretation.

Since the match results in this study are multi-class (win, draw, loss), a 3 × 3 matrix is used to depict the correspondence between the three true labels and the three predicted labels. The confusion matrix for the three-class prediction results (see Table 2) is shown, where N_ij_ represents the number of samples where the actual class is i and the predicted class is j. W, D, and L represent win, draw, and loss, respectively, and the total number of samples is $N=\sum_{i}\sum_{j}\;N_{\mathrm{ij}}$ . In the three-class scenario, this study used the One-vs-Rest (OvR) approach to construct a binary classification view for each class. For the “win (W)” case, the true positive (TPW) is defined as the actual win and predicted win: ${\mathrm{TP}}_{W}=N_{\mathrm{WW}}$ . The false positive (FPW) is defined as the actual draw or loss but predicted as win: ${\mathrm{FP}}_{W}=N_{\mathrm{DW}}+N_{\mathrm{LW}}$ . The false negative (FNW) is defined as the actual win but predicted as draw or loss: ${\mathrm{FN}}_{W}=N_{\mathrm{WD}}+N_{\mathrm{WL}}$ . The true negative (TNW) is the remaining samples: ${\mathrm{TN}}_{W}=N-{\mathrm{TP}}_{W}-{\mathrm{FP}}_{W}-{\mathrm{FN}}_{W}$ . The same approach is applied for “draw (D)” and “loss (L)” to obtain ${\mathrm{TP}}_{D}=N_{\mathrm{DD}}$ , ${\mathrm{FP}}_{D}=N_{\mathrm{WD}}+N_{\mathrm{LD}}$ , ${\mathrm{FN}}_{D}=N_{\mathrm{DW}}+N_{\mathrm{DL}}$ , and ${\mathrm{TP}}_{L}=N_{\mathrm{LL}}$ , ${\mathrm{FP}}_{L}=N_{\mathrm{WL}}+N_{\mathrm{DL}},{\mathrm{FN}}_{L}={\mathrm{NL}}_{W}+N_{\mathrm{LD}}$ .

$$\text{Accuracy}=\frac{\;(N_{\mathrm{WW}}+N_{\mathrm{DD}}+N_{\mathrm{LL}})\;}{N}$$ (1)

$$\text{Precisionc}=\frac{\mathrm{TPc}\;}{\;(\mathrm{TPc}+\mathrm{FPc})}$$ (2)

$$\text{Recallc}=\frac{\mathrm{TPc}\;}{\;(\mathrm{TPc}+\mathrm{FNc})}$$ (3)

$$F1c=\frac{2\times \text{Precisionc}\times \text{Recallc}\;}{\;(\text{Precisionc}+\text{Recallc})}\;$$ (4)

**Table 2:** Three-class confusion matrix (win-draw-loss).

Match outcome	Predicted win	Predicted draw	Predicted loss
Actual Win	NWW	NWD	NWL
Actual Draw	NDW	NDD	NDL
Actual Loss	NLW	NLD	NLL

Report the macro-average or weighted macro-average metrics $F1_{\text{macro}}=\frac{1}{3}\;(F1_{W}+F1_{D}+F1_{L})\;$ or $F1_{\text{weighted}}=\sum \_c\;\frac{({\text{support}}_{c}\;)\;}{N}\cdot F1_{c}$ .

**Figure 2:**
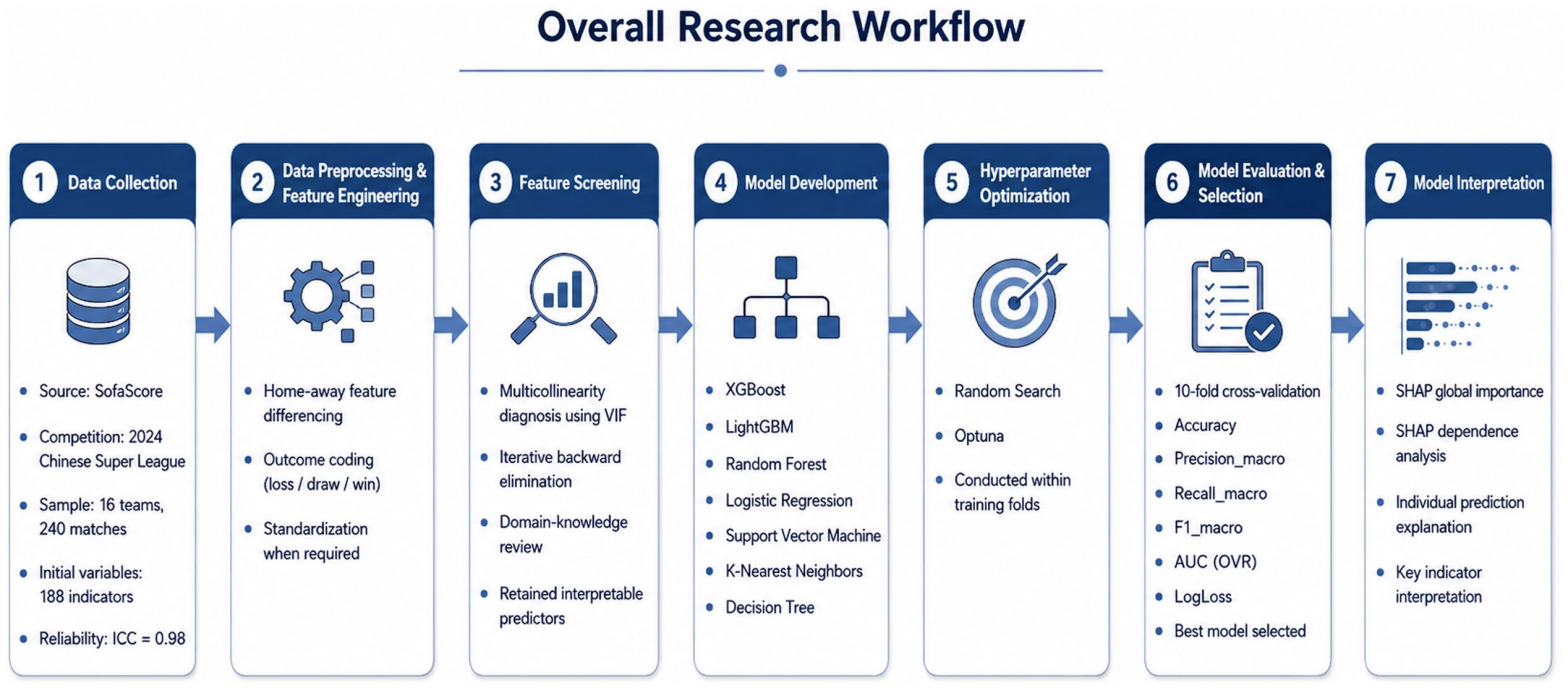
Overall research workflow of the explainable machine learning analysis. The workflow includes data collection, data preprocessing and feature engineering, feature screening, model development, hyperparameter optimization, model evaluation and selection, and SHAP-based model interpretation.

All code was implemented in Python, and the Scikit-learn library was used for model development, training, and evaluation. This ensured the consistent implementation of the machine learning algorithms and analytical procedures used in this study. The proposed machine learning workflow is shown in Figure 2.

## 4 Results

### 4.1 Model results

Within the stratified 10-fold cross-validation framework conducted on the training set, this study compared seven machine learning models and evaluated their performance using a held-out validation set containing samples not involved in model training. The validation results showed that distinguishing home win, draw, and home loss from single-season post-match aggregate data remained a challenging three-class classification task, consistent with the complex and context-dependent nature of football match outcomes (Oliva-Lozano et al., 2025). RandomForest-RandomizedSearchCV achieved the highest macro-F1 score on the held-out validation set (0.538), but its training-set macro-F1 was close to 1.00 and was markedly higher than its cross-validation and held-out validation results. This pattern may be related to the limited sample size, the three-class structure of the outcome variable, the relatively ambiguous boundary of the draw class, and the difficulty of modelling football match outcomes using single-season post-match aggregate indicators.

LightGBM-Optuna achieved a slightly higher AUC-OVR than XGBoost-Optuna on the held-out validation set, indicating comparable class-discrimination ability. Its Accuracy and macro-F1 were slightly lower than those of XGBoost-Optuna. As a gradient boosting decision tree model, LightGBM has advantages in training efficiency and nonlinear modelling capability (Ke et al., 2017). Logistic Regression, SVM, KNN, and Decision Tree showed relatively limited validation performance under the current sample size and feature structure. KNN and the single Decision Tree model produced lower macro-F1 scores, while Logistic Regression showed higher LogLoss under certain tuning settings, indicating less stable probability estimation.

The LogLoss results provided complementary information on the probabilistic quality of the classifiers. In contrast to Accuracy and macro-F1, which are based on discrete class assignments, LogLoss evaluates the predicted probability distribution and penalizes confident probability assignments to incorrect classes (Gneiting and Raftery, 2007). The relatively large LogLoss values reported in Table 3 indicate that classification performance and probability calibration were not fully aligned. In other words, a model may produce a moderate Accuracy or macro-F1 score while still assigning high confidence to incorrect classifications. These results suggest that the predicted probabilities should be interpreted cautiously, particularly under the limited sample size and three-class outcome structure of the present study.

**Table 3:** Validation-set performance results of each model after hyperparameter tuning.

Model	Tuning	Accuracy	Precision_macro	Recall_macro	F1_macro	AUC_OVR	LogLoss
KNN	RandomizedSearchCV	0.375	0.322	0.324	0.319	0.513	1.868
	Optuna	0.417	0.367	0.362	0.357	0.528	1.854
SVM	RandomizedSearchCV	0.479	0.467	0.451	0.455	0.668	1.077
	Optuna	0.500	0.481	0.473	0.474	0.650	1.059
RandomForest	RandomizedSearchCV	0.583	0.540	0.547	0.538	0.670	0.989
	Optuna	0.542	0.519	0.518	0.517	0.718	0.952
DecisionTree	RandomizedSearchCV	0.500	0.486	0.487	0.468	0.619	15.862
	Optuna	0.458	0.417	0.427	0.419	0.573	8.177
XGBoost	RandomizedSearchCV	0.542	0.474	0.481	0.476	0.743	0.877
	Optuna	0.604	0.529	0.547	0.535	0.739	0.898
LightGBM	RandomizedSearchCV	0.521	0.531	0.526	0.515	0.729	1.010
	Optuna	0.562	0.525	0.525	0.523	0.743	0.904
LogisticRegression	RandomizedSearchCV	0.479	0.412	0.427	0.416	0.645	2.178
	Optuna	0.438	0.388	0.383	0.383	0.606	3.477

SVM and KNN are classical classification algorithms, but their performance in the present dataset may have been sensitive to feature-space distribution, sample size, and parameter settings (Kecman, 2005; Cover and Hart, 1967). XGBoost-Optuna achieved the highest held-out validation Accuracy among the compared models (0.604), with a macro-F1 of 0.535 and an AUC-OVR of 0.739. Its macro-F1 was close to the best result obtained by RandomForest-RandomizedSearchCV. Despite the difference between training and validation performance, XGBoost-Optuna showed competitive validation performance, relatively stable probabilistic output, and tree-based interpretability within the current model-comparison framework. It was therefore used as the base model for the subsequent SHAP analysis (Chen and Guestrin, 2016; Lundberg and Lee, 2017).

### 4.2 Interpretability

#### 4.2.1 Global and class-specific SHAP feature contribution analysis

To examine the relative contribution of each feature in the XGBoost model, SHAP values were calculated for the three-class classification model. SHAP values were used to characterize both the direction and magnitude of each variable’s contribution to the model output for the corresponding class. For a given outcome class, a positive SHAP value indicates that the variable increases the model’s tendency to classify a sample into that class, whereas a negative SHAP value indicates that the variable decreases the model output for that class. It should be noted that SHAP analysis reflects the contribution of variables to model output, rather than the causal effect of variables on match outcomes.

**Figure 3:**
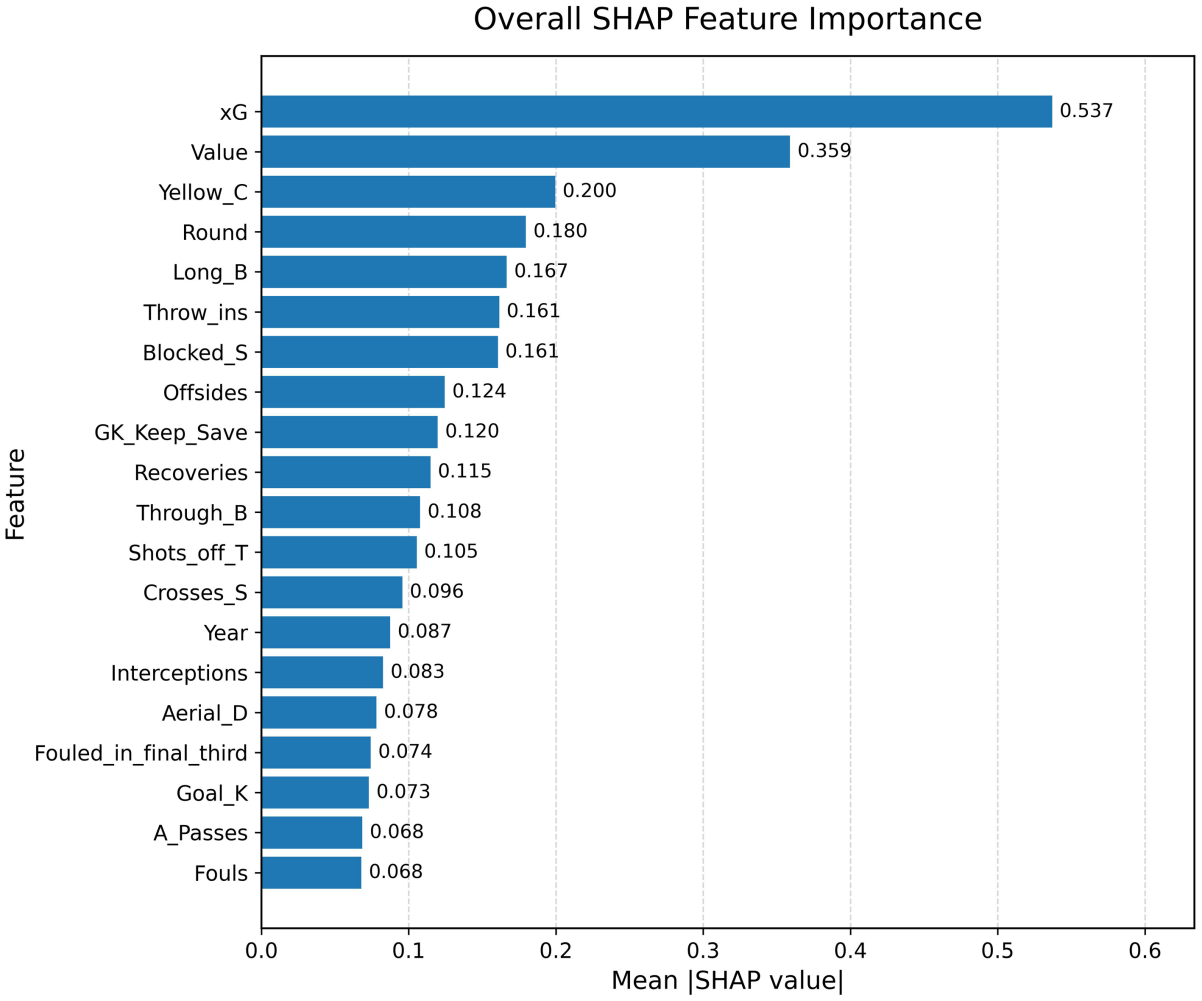
Overall SHAP feature importance ranking. The horizontal bars represent the mean absolute SHAP values of the top-ranked features, indicating their relative contribution to the overall model output.

The global SHAP feature importance results showed that xG was the variable with the highest model contribution, with a mean absolute SHAP value of 0.537; Value ranked second, with a mean absolute SHAP value of 0.359 (Figure 3). The contribution magnitudes of these two variables were clearly higher than those of the other variables, indicating that the model mainly used information related to attacking chance quality and player attributes during the overall classification process. Yellow_C, Round, Long_B, Throw_ins, and Blocked_S subsequently ranked among the most important features, with mean absolute SHAP values of 0.200, 0.180, 0.167, 0.161, and 0.161, respectively. Offsides, GK_Keep_Save, Recoveries, Through_B, Shots_off_T, Crosses_S, Year, Interceptions, and Aerial_D also entered the top 20 variables in the global importance ranking, although their overall contribution magnitudes were lower than those of xG and Value.

**Figure 4:**
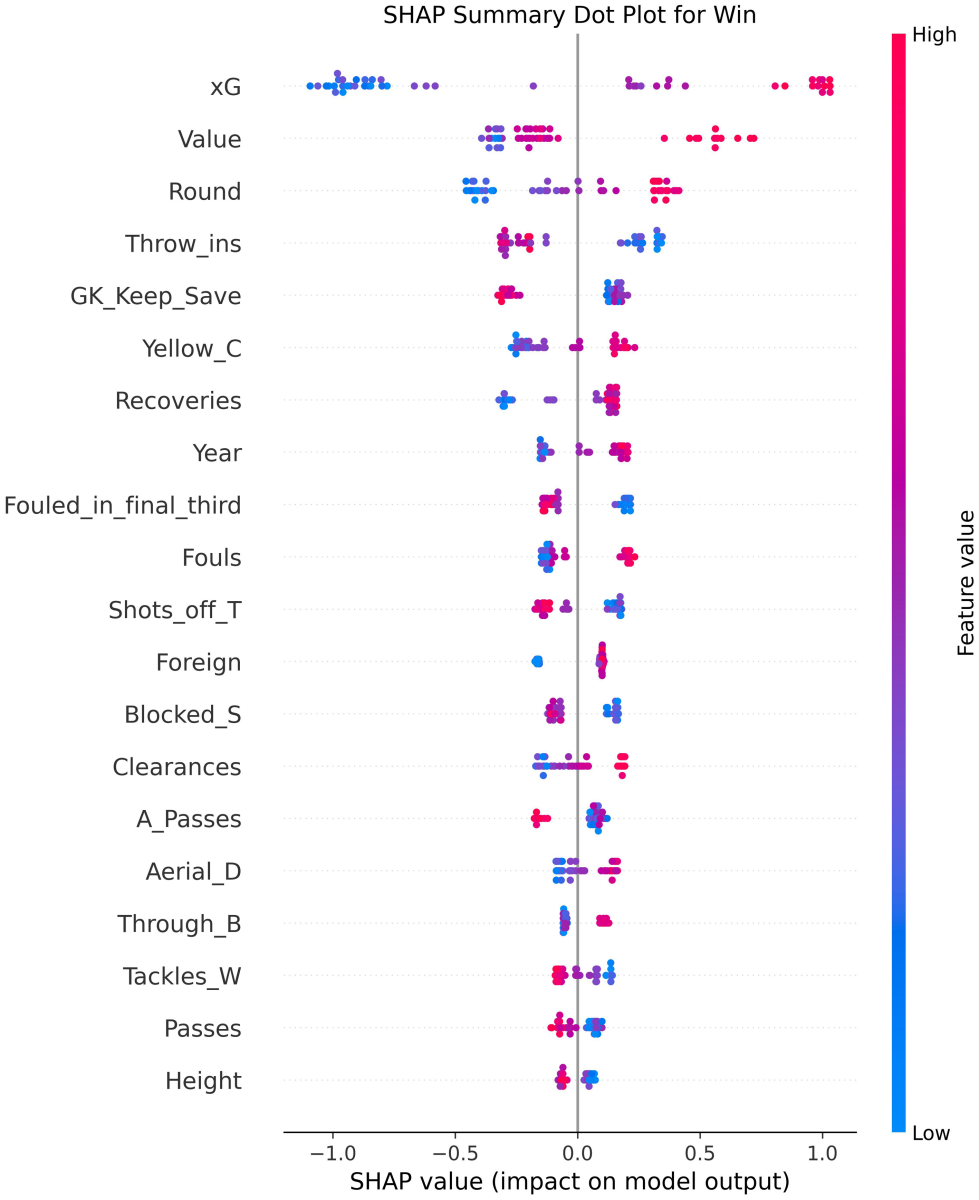
SHAP summary plot for the home win class. Each point represents the SHAP value of one sample for a given feature, and the color indicates the corresponding feature value.

**Figure 5:**
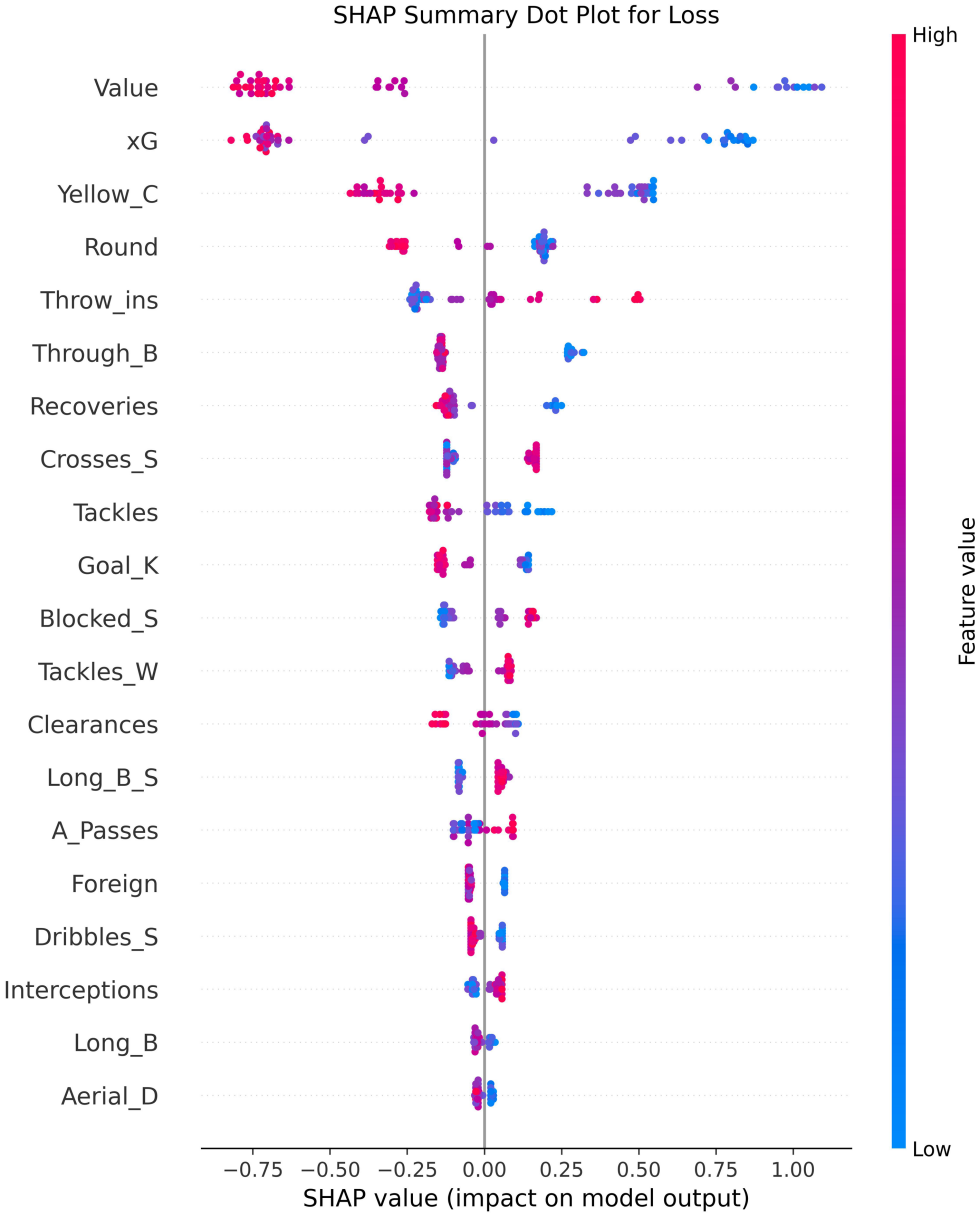
SHAP summary plot for the home loss class. Each point represents the SHAP value of one sample for a given feature, and the color indicates the corresponding feature value.

**Figure 6:**
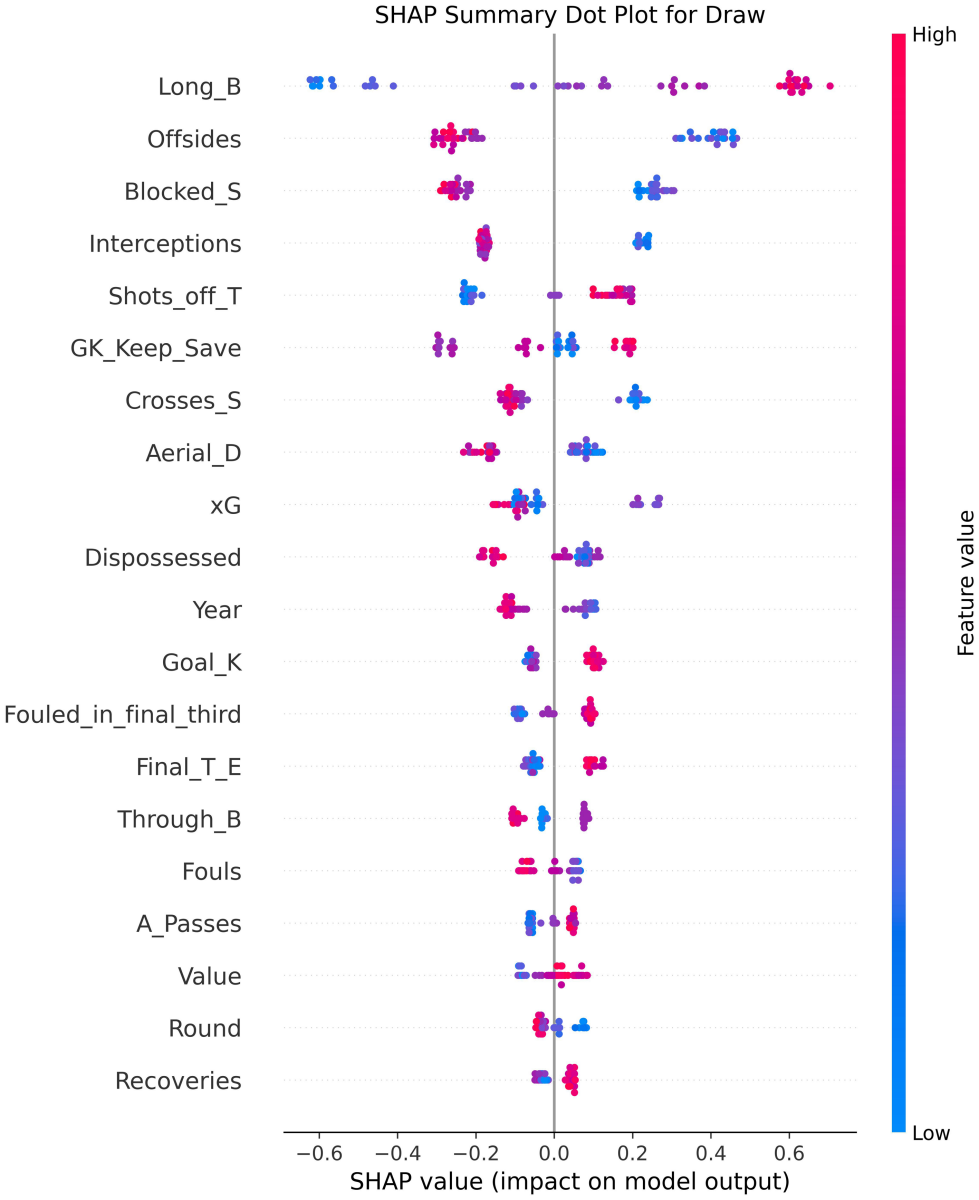
SHAP summary plot for the draw class. Each point represents the SHAP value of one sample for a given feature, and the color indicates the corresponding feature value.

The class-specific SHAP summary plots showed differences in the contribution directions and distribution ranges of variables across outcome classes (Figures 4–6). In the home win class, xG and Value remained among the highest-ranking variables (Figure 4). Higher xG values were mainly distributed in the positive SHAP region, whereas lower xG values were mainly distributed in the negative SHAP region; Value showed a similar distribution pattern. In addition to xG and Value, Round, Throw_ins, GK_Keep_Save, Yellow_C, Recoveries, Year, Fouled_in_final_third, Fouls, and Shots_off_T also showed certain model contributions in the home win class, although their SHAP distribution ranges were generally smaller than those of xG and Value.

In the home loss class, Value, xG, Yellow_C, Round, and Throw_ins ranked among the leading contributors (Figure 5). Compared with the home win class, the SHAP directions of xG and Value were generally reversed; that is, higher values were more often distributed in the negative SHAP region, whereas lower values were more often distributed in the positive SHAP region. Through_B, Recoveries, Crosses_S, Tackles, Goal_K, Blocked_S, and Tackles_W also entered the important variable ranking for this class, but their contribution magnitudes were relatively limited.

In the draw class, SHAP values for all variables were more concentrated around zero, and the contribution magnitude of individual variables was smaller than that observed in the home win and home loss classes (Figure 6). Long_B, Offsides, Blocked_S, Interceptions, and Shots_off_T ranked relatively high in this class, with Long_B showing a relatively wide SHAP distribution range. GK_Keep_Save, Crosses_S, Aerial_D, xG, Dispossessed, Year, and Goal_K also entered the important variable ranking for the draw class; however, most variables still had SHAP values concentrated around zero and did not show the same degree of directional distribution as observed in the home win and home loss classes.

#### 4.2.2 SHAP dependence plots and model-derived transition points

To further describe the nonlinear model contribution patterns of important variables in the home win class, SHAP univariate dependence plots were generated for the home win class. In these plots, each scatter point represents the SHAP value of an individual sample for the corresponding variable, while the smoothed curve shows the overall trend between the variable value and its SHAP contribution. The vertical dashed line indicates the model-derived potential transition point, and the shaded area represents the bootstrap 95% confidence interval.

**Figure 7:**
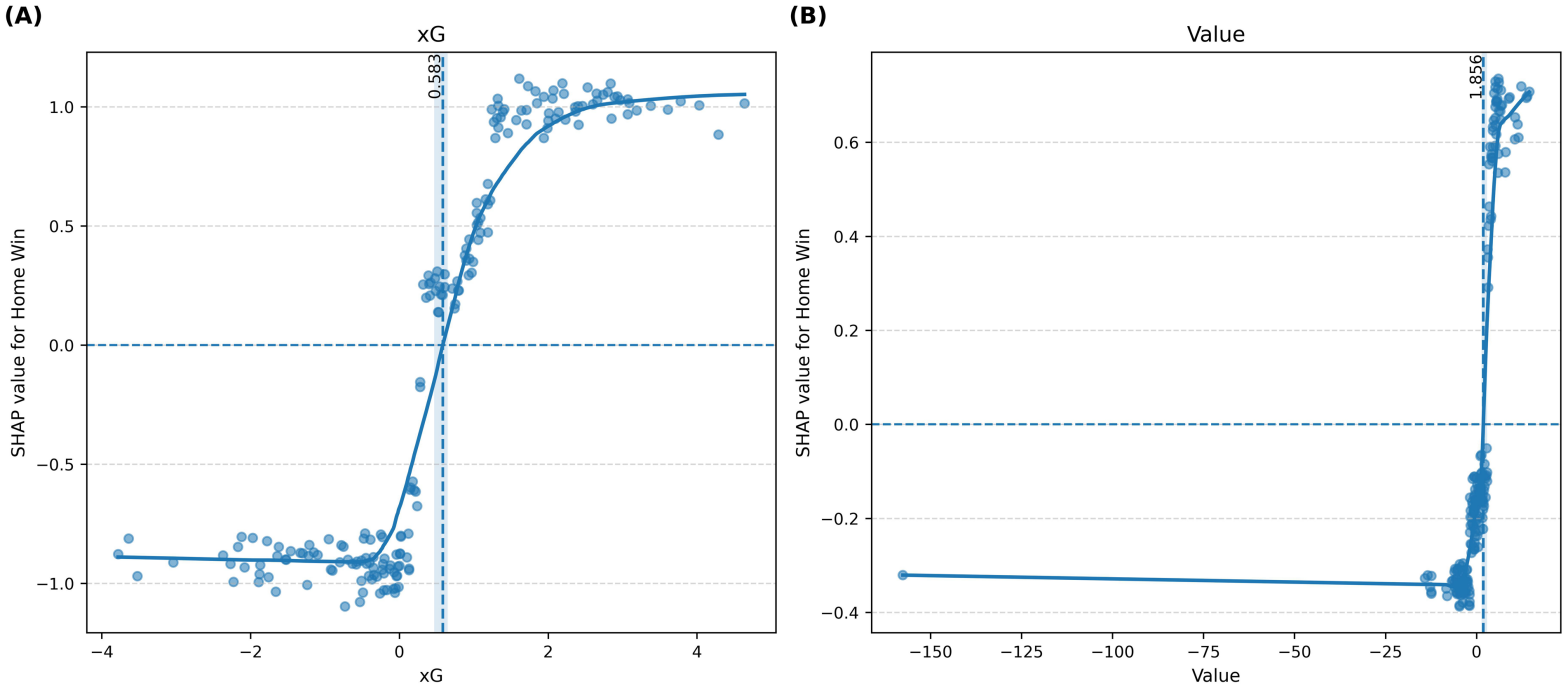
SHAP dependence plots of xG and Value for the home win class. The smoothed curves show the nonlinear contribution patterns of the variables, and the vertical dashed lines indicate the model-derived transition points.

**Figure 8:**
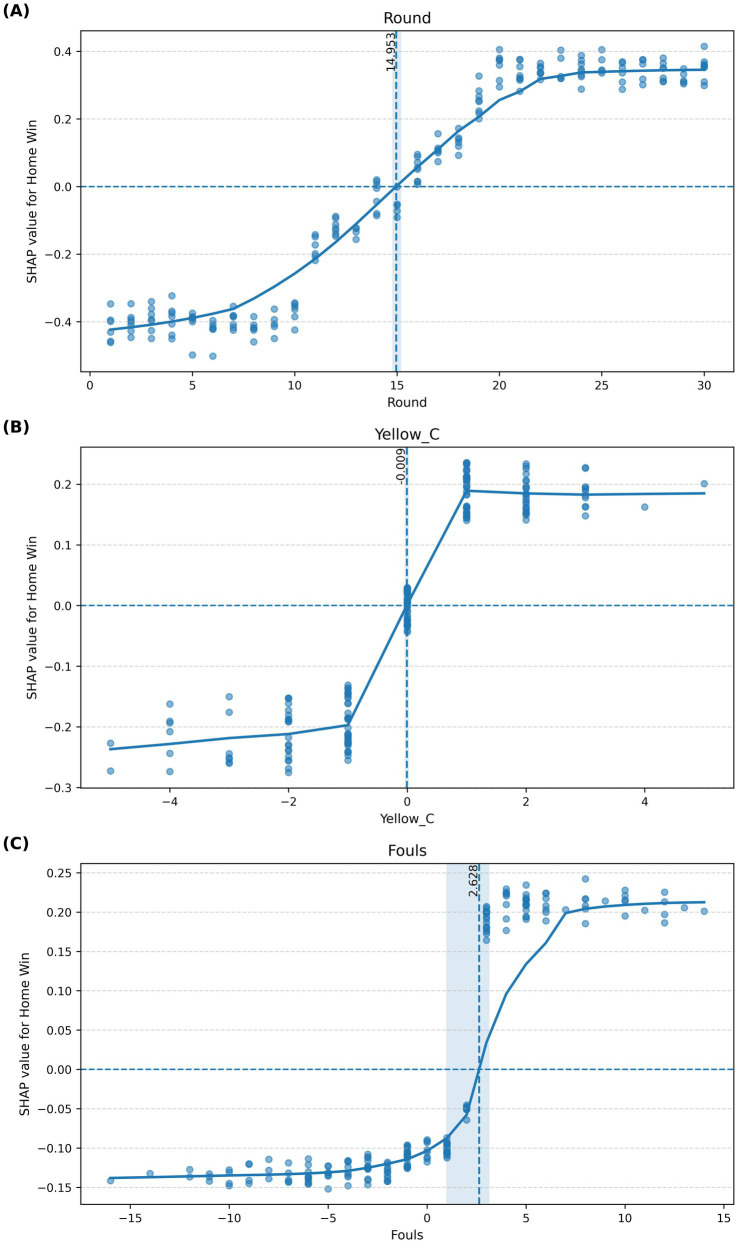
SHAP dependence plots of Round, Yellow_C, and Fouls for the home win class. The smoothed curves show the nonlinear contribution patterns of the variables, and the vertical dashed lines indicate the model-derived transition points.

**Figure 9:**
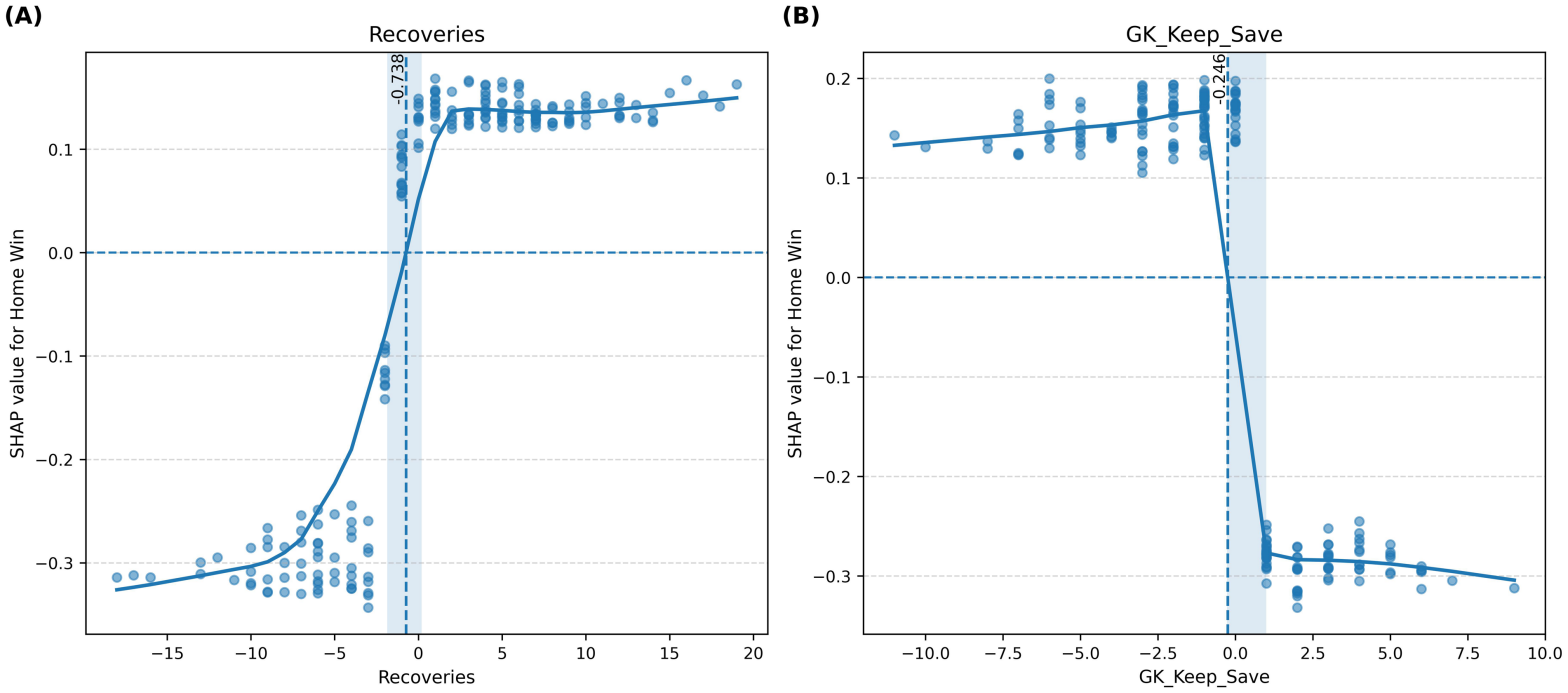
SHAP dependence plots of Recoveries and GK Keep Save for the home win class. The smoothed curves show the nonlinear contribution patterns of the variables, and the vertical dashed lines indicate the model-derived transition points.

**Figure 10:**
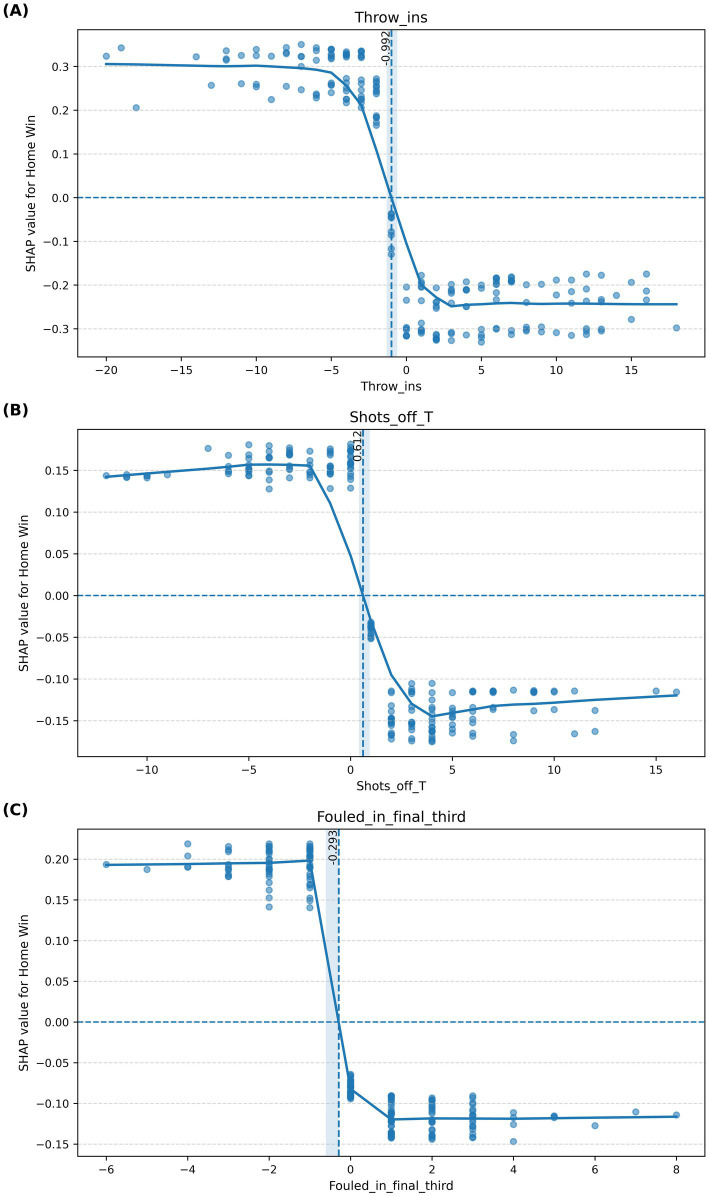
SHAP dependence plots of Throw ins, Shots off T, and Fouled in final third for the home win class. The smoothed curves show the nonlinear contribution patterns of the variables, and the vertical dashed lines indicate the model-derived transition points.

From the overall trend, Figures 7–10 show that several variables in the home win class did not exhibit a simple linear contribution pattern. Instead, changes in contribution direction or contribution magnitude occurred around specific ranges of model input values. Specifically, after exceeding the model-derived transition points, the SHAP values of xG, Value, Round, Yellow_C, Fouls, and Recoveries generally shifted from negative to positive. In contrast, GK_Keep_Save, Throw_ins, Shots_off_T, and Fouled_in_final_third showed a shift from positive to negative.

Figure 7 shows that both xG and Value exhibited relatively clear negative-to-positive transitions. The smoothed curve for xG crossed the zero-contribution baseline at approximately 0.583. Below this transition point, sample SHAP values were mainly distributed in the negative region; after exceeding this range, SHAP values rapidly shifted into the positive region and gradually tended to plateau at higher values (Figure 7A). The model-derived transition point for Value was approximately 1.856, and the curve increased rapidly around this range. SHAP values in the lower-value range were mainly concentrated in the negative region, whereas those in the higher-value range were more frequently distributed in the positive region (Figure 7B). These results indicate that, in the current model, both xG and Value showed a change in contribution direction around specific input ranges.

Figure 8 further shows that Round, Yellow_C, and Fouls also exhibited negative-to-positive SHAP dependence patterns. The model-derived transition point for Round was approximately 14.953, where the curve crossed the zero-contribution baseline. SHAP values were mainly negative in earlier rounds, then gradually shifted to positive after exceeding this range and remained at a relatively stable positive level in later rounds (Figure 8A). The transition point for Yellow_C was close to zero, at approximately −0.009. The curve shifted from negative to positive around this range and then remained relatively stable at higher values (Figure 8B). The transition point for Fouls was approximately 2.628. Around this value, the smoothed curve moved from the negative SHAP region into the positive region, and then gradually tended to plateau at higher values (Figure 8C).

In Figure 9, Recoveries and GK_Keep_Save showed opposite changes in contribution direction. The model-derived transition point for Recoveries was approximately −0.738. Below this transition point, SHAP values were mainly concentrated in the negative region; after exceeding this range, they rapidly shifted to positive and remained relatively stable at higher values (Figure 9A). The transition point for GK_Keep_Save was approximately −0.246, but its direction of change was opposite to that of Recoveries. Around this range, the curve rapidly shifted from positive to negative. When the variable value exceeded this range, sample SHAP values were mainly distributed in the negative region and remained at a relatively low level across the subsequent value range (Figure 9B).

Figure 10 shows positive-to-negative transition patterns for Throw_ins, Shots_off_T, and Fouled_in_final_third. The model-derived transition point for Throw_ins was approximately −0.992. Around this range, the curve rapidly moved from the positive SHAP region into the negative region and remained at a relatively stable negative level at higher values (Figure 10A). The transition point for Shots_off_T was approximately −0.612, and its smoothed curve similarly shifted from positive to negative near zero, then maintained a negative SHAP distribution at higher values (Figure 10B). The transition point for Fouled_in_final_third was approximately 0.293. Around this range, the curve shifted from the positive region into the negative region and maintained a relatively stable negative contribution after exceeding this value (Figure 10C).

The SHAP dependence plots for the home win class show that the contributions of some variables in the model did not simply accumulate linearly as their values increased. Instead, direction changes or contribution stabilization occurred around specific input ranges. xG, Value, Round, Yellow_C, Fouls, and Recoveries generally shifted to positive SHAP contributions after exceeding their corresponding transition points, whereas GK_Keep_Save, Throw_ins, Shots_off_T, and Fouled_in_final_third generally shifted to negative SHAP contributions after exceeding their corresponding transition points. These results reflect only potential threshold-like SHAP patterns at the model level and provide a basis for the subsequent discussion of the model contribution characteristics of different variables in match outcome classification.

#### 4.2.3 Exploratory consistency assessment of model-derived transition points

To avoid interpreting potential threshold-like patterns based solely on SHAP dependence plots, an exploratory consistency assessment was further conducted using the 10 model-derived transition points shown in Figures 7–10 as cut-off values. This analysis examined whether changes in the home win proportion on both sides of each transition point were consistent with the direction of the SHAP curve. Samples were divided into low-value and high-value groups according to the model-derived transition point of each variable, and the direction of change in the home win proportion was compared between the two groups. The results showed that, for 8 of the 10 variables, the direction of change in the home win proportion on both sides of the transition point was consistent with the direction of the SHAP dependence curve.

For variables whose SHAP curves shifted from negative to positive, the home win proportion was higher in the high-value group than in the low-value group. Specifically, the home win proportions in the low- and high-value groups were 27.2 and 71.2% for xG, 34.0 and 71.8% for Value, 37.5 and 53.9% for Round, 36.4 and 53.2% for Yellow_C, 40.6 and 57.5% for Fouls, and 29.3 and 58.2% for Recoveries, respectively. Among these variables, xG, Value, and Recoveries showed relatively larger between-group differences, with Cramer’s V values of 0.438, 0.381, and 0.286, respectively. For variables whose SHAP curves shifted from positive to negative, the raw-data grouping results for GK_Keep_Save and Throw_ins were also consistent with the SHAP curve direction. Samples above the model-derived transition point showed lower home win proportions: GK_Keep_Save decreased from 56.7 to 35.8%, and Throw_ins decreased from 55.8 to 37.8%. However, Shots_off_T and Fouled_in_final_third showed inconsistent directions in the home win proportion on both sides of the transition point, and their between-group differences were relatively weak.

## 5 Discussion

### 5.1 Model interpretation

Match-related technical statistics can effectively distinguish winning, drawing, and losing teams (Lago-Peñas et al., 2010; Liu et al., 2015; Castellano et al., 2012). The indicators were classified into player attributes, match context, attacking performance, possession and passing performance, duels and contests, defensive performance, and disciplinary performance (Pena and Touchette, 2012; Liu et al., 2015; Kessouri, 2023). Combined with the SHAP results, the model’s discriminative basis was not concentrated in a single dimension (Lago-Peñas et al., 2010). Instead, it showed a structure in which attacking quality and squad resources served as the main sources of information, while match context, progression patterns, defensive events, and disciplinary performance provided complementary information (Plakias et al., 2024). xG ranked first in the global SHAP importance results. It is not merely a substitute for the number of shots, but is closer to chance quality itself, as it can reflect shot location, chance type, and scoring probability (Rathke, 2017). In the home win class, higher xG values were mostly located in the positive SHAP region; in the home loss class, higher xG values more often corresponded to negative contributions. This indicates that the model clearly relied on the difference in chance quality between the home and away teams when distinguishing win and loss outcomes (Liu et al., 2015; Rathke, 2017). Because xG is derived from the match process and is closely linked to the final match outcome, its high contribution in the model is more appropriately understood as a core explanatory signal for post-match review. For coaches and analysts, the key issue is to examine where high-quality chances came from: effective progression, high pressing, set pieces, wide crosses, or defensive errors by the opponent. If a team had an xG advantage but failed to win, the analysis may shift toward finishing efficiency, the handling of key shots, and goalkeeper performance (Mitrotasios et al., 2025). If a team won despite not having an xG advantage, video review can be used to examine whether the result was related to individual player ability, while further checking the role of key events and a small number of high-value chances. Value ranked second in the global SHAP importance results. It belongs to the player attributes dimension and is not a specific technical or tactical action, but it provides important contextual information in the model and may offer a reference for subsequent recruitment evaluation. In the home win class, higher Value was more likely to correspond to positive SHAP values; in the home loss class, higher Value more often showed negative contributions. This indicates that the model captured differences in squad resources when distinguishing win and loss outcomes. Value is better understood as a composite signal reflecting team resources, player ability structure, and the overall competitive foundation (Constantinou and Fenton, 2013). A single match can still be affected by opposition quality, tactical arrangements, match-day form, injuries, and key events. The value of this variable is that it reminds us that match performance evaluation should not be separated from the resource structure of the team.

Yellow_C, Round, Long_B, Throw_ins, Blocked_S, GK_Keep_Save, and Recoveries ranked among the leading variables in the global importance results, indicating that the model interpretation was not limited to attacking indicators. Round is a match context variable that may contain information related to season stage, accumulated team form, ranking pressure, and fixture context. Yellow_C and Fouls reflect disciplinary performance and contest intensity. Long_B, Throw_ins, Through_B, and A_Passes are more closely related to possession and progression patterns (Lago, 2009). Blocked_S, GK_Keep_Save, Recoveries, Interceptions, and Clearances are related to defensive pressure, ball recovery, and attacking–defensive transitions.

The presence of these variables indicates that model classification of CSL match outcomes cannot be explained by “attacking quality” alone. Although chance quality was the strongest signal, match context, progression patterns, and defensive processes also contributed to class differentiation (Oliva-Lozano et al., 2025). Variables such as Throw_ins, Long_B, and GK_Keep_Save should not be interpreted simply as “the more, the better.” Throw-ins and long balls may indicate active progression, but they may also suggest that ground-based possession was being disrupted (Bradley et al., 2013). Goalkeeper saves may reflect individual goalkeeper ability, but they may also indicate that the team was under prolonged defensive pressure. Once statistical indicators are separated from the match context, their explanatory value is substantially reduced.

The SHAP distribution for the draw class was more concentrated around zero. Long_B, Offsides, Blocked_S, Interceptions, Shots_off_T, GK_Keep_Save, Crosses_S, and Aerial_D ranked relatively high in the draw class, but no dominant variables comparable to xG and Value in the home win and home loss classes emerged. Draws were more likely to reflect the combined effect of multiple weak signals. When neither team establishes a sufficient advantage in chance quality, progression efficiency, defensive pressure, or key events, the match outcome is more likely to remain balanced. In reviewing drawn matches, focusing only on xG or shots is insufficient; the analysis should return to the match process and examine goal timing, attacking–defensive transitions, key refereeing decisions, and changes in match tempo.

### 5.2 Interpretive boundaries of model-derived transition points

SHAP dependence plots showed that some variables in the home win class did not exhibit simple linear contributions. After exceeding the model-derived transition points, the SHAP values of xG, Value, Round, Yellow_C, Fouls, and Recoveries generally shifted from negative to positive, whereas GK_Keep_Save, Throw_ins, Shots_off_T, and Fouled_in_final_third showed a shift from positive to negative. When processing these variables, the model did not simply equate higher indicator values with a higher probability of home win; instead, the contribution to the home win class changed around specific input ranges. SHAP is essentially used to explain feature contributions to model output, rather than to directly prove causal effects of variables on real match outcomes (Lundberg and Lee, 2017; Molnar, 2020).

From the perspective of post-match review, the transitions in xG and Value indicate that the model was relatively sensitive to advantages in chance quality and differences in squad resources when identifying home win samples. For the coaching staff, this does not mean that reaching a specific xG value or player market value can guarantee victory. Rather, post-match review should focus on how high-quality chances were created, at which stage of the attacking sequence they occurred, which players were involved in the finishing phase, how they handled those situations, and whether squad resources were effectively transformed into on-field advantages (Lago-Peñas et al., 2010; Liu et al., 2015).

The transition points for Round, Yellow_C, and Fouls more strongly reflected information related to match context and match intensity. The transition observed around the middle of the season for Round suggests that the model used information embedded in the season stage, including team form, fixture context, and competitive background. The contribution changes in Yellow_C and Fouls should not be interpreted as evidence that yellow cards or fouls are beneficial for winning. Rather, disciplinary events and foul differences may carry information about contest intensity, match tempo, and the degree of on-field control in the model (Lago, 2009). In actual post-match review, these indicators should be interpreted in relation to the location and timing of fouls, whether they disrupted the opponent’s transition attack, whether they created set-piece risks, and whether the subsequent phase developed into a threatening attack, rather than being evaluated only by their frequency.

The opposite contribution directions of Recoveries and GK_Keep_Save further highlight the value of model interpretation. After Recoveries exceeded the model-derived transition point, its contribution shifted to the positive region, indicating that ball recovery information made a relatively stable model contribution to the home win class (Low et al., 2021). In practical analysis, this finding can guide analysts to focus their video review on the quality of counter-pressing after losing possession, the speed at which second-phase attacks are created, and whether midfield and front-line pressing generated effective threats. In contrast, GK_Keep_Save entered the negative contribution region after exceeding its transition point. An increase in goalkeeper saves does not necessarily indicate better defensive quality; it may also suggest insufficient defensive protection, repeated pressure in the penalty area, or more high-quality shots conceded to the opponent. Therefore, goalkeeper-save data should be interpreted together with the opponent’s shot location, shot quality, defensive line positioning, and penalty-area protection (Tienza-Valverde et al., 2023).

The negative transitions in Throw_ins, Shots_off_T, and Fouled_in_final_third also remind us that attacking-process variables should not be judged solely by their frequency. An increase in throw-ins may result from wide progression, but it may also indicate that attacks were frequently interrupted. More shots off target do not necessarily mean better attacking quality. A higher number of fouls won in the final third may suggest that the team had stronger access to advanced areas, but it may also indicate insufficient subsequent set-piece execution or attacking conversion. For match analysts, the value of these variables does not lie in making a direct positive or negative judgment (Bradley et al., 2013; Stone et al., 2025). Rather, they help indicate which match clips should be reviewed to determine whether these events actually generated effective attacking threats.

### 5.3 Limitations and future outlook

This study has several limitations. The sample was limited to the 2024 CSL season, including 240 matches. Single-season data are likely to be affected by fixture scheduling, team form, foreign-player configuration, injuries, coaching changes, and season stage. Therefore, the feature contribution patterns identified by the current model still need to be further examined using multi-season data, samples from different leagues, and external datasets. At the same time, the same team appeared repeatedly within a single season, which may have introduced team-level correlations between match samples, meaning that the observations were not fully independent. The current model did not explicitly include team random effects or a team-level hierarchical structure, and therefore could not fully control for the influence of team-specific characteristics on the model results. Future studies may adopt cross-season validation, external validation sets, or team-grouped cross-validation strategies to further examine the stability of the model interpretation results.

This study used post-match aggregate statistics. Variables such as xG, shots, goalkeeper saves, and Recoveries were all derived from the match process or post-match statistics. Post-match aggregate data cannot fully represent the complete match process. For example, information on how attacking chances were created, which areas generated defensive pressure, in what situations ball recoveries occurred, and at what match stage yellow cards were shown cannot be directly obtained from aggregate indicators. Therefore, future research should combine event-level data, positional tracking data, running-load data, and video annotation data, so that the high-contribution variables identified by the model can be reinterpreted within specific match contexts.

Although this study used within-training-set cross-validation, held-out validation-set evaluation, and a Pipeline workflow to minimize the risk of data leakage, model performance may still be affected by the training–validation split, class distribution, and hyperparameter search space because of the limited sample size and the inherent difficulty of the three-class classification task. The draw class had a relatively small number of samples and a more ambiguous feature boundary, which may have further increased the difficulty of model identification for this class. The model-derived transition points in this study were obtained from SHAP dependence curves and were further supported by bootstrap confidence intervals and the raw-data consistency assessment. This approach is more robust than simple visual inspection, but it still belongs to model interpretation and descriptive analysis, and should not be regarded as strict causal threshold validation. Future studies seeking to further confirm these nonlinear patterns may use segmented regression, generalized additive models, change-point detection, partial dependence plots, or accumulated local effects analysis, and repeat the analysis using independent samples.

Future research may further integrate event-level match processes, positional tracking data, video annotation, and psychological measurement data on the basis of post-match performance data, so as to interpret the high-contribution indicators identified by the model within more detailed match contexts. This would not only improve the stability of model interpretation, but also help translate post-match statistical results into more specific technical and tactical analytical evidence.

## 6 Conclusion

Based on post-match data from 240 matches in the 2024 CSL season, this study constructed an explainable machine learning framework to examine how technical, tactical, contextual, and player-attribute indicators contributed to the classification of home win, draw, and home loss. After comparing multiple machine learning models, XGBoost was selected as the base model for SHAP analysis because of its comparatively balanced validation performance and tree-based interpretability.

The SHAP results showed that xG and Value had the highest model contributions, indicating that chance quality and squad-resource-related information were the main signals used by the model to distinguish home win and home loss outcomes. Other indicators, including Yellow_C, Round, Long_B, Throw_ins, Blocked_S, GK_Keep_Save, and Recoveries, also contributed to the classification process, suggesting that match outcome classification was informed by multidimensional information related to match context, possession progression, defensive events, goalkeeper involvement, and disciplinary performance. Class-specific SHAP results further showed clearer contribution directions for the home win and home loss classes, whereas the draw class showed weaker dominance of individual variables.

Several variables exhibited potential nonlinear contribution patterns and model-derived transition points in the home win class. The raw-data consistency assessment provided descriptive support for most of these model-derived patterns, but these findings should be interpreted as model-level explanatory evidence rather than causal thresholds. Overall, this study provides a data-driven reference for contextual video review, performance diagnosis, and training-priority development in the CSL. Future studies should further examine the stability and practical value of these findings using multi-season samples, event-level data, positional tracking data, and external validation.

## Data Availability

Publicly available datasets were analyzed in this study. This data can be found at: sofascore.
